# The Effect of Smoking on the Activated Clotting Time and the Incidence of Complications in Noncardiac Arterial Procedures

**DOI:** 10.1177/15266028231207027

**Published:** 2023-10-27

**Authors:** Liliane C. Roosendaal, Tristan E. K. van Os, N. van Es, M. Hoebink, Arno M. Wiersema, Jan D. Blankensteijn, Vincent Jongkind

**Affiliations:** 1 Department of Vascular Surgery, Dijklander Ziekenhuis, Hoorn, The Netherlands; 2 Department of Vascular Surgery, Amsterdam University Medical Center, VUmc, Amsterdam, The Netherlands; 3 Microcirculation, Amsterdam Cardiovascular Sciences, Amsterdam, The Netherlands; 4 Department of Vascular Medicine, Amsterdam University Medical Center, Amsterdam, The Netherlands; 5 Pulmonary Hypertension & Thrombosis, Amsterdam Cardiovascular Sciences, Amsterdam, The Netherlands

**Keywords:** smoking, heparin, activated clotting time, vascular surgical procedures

## Abstract

**Purpose:**

Smoking is a well-known risk factor for developing arterial diseases and for an increase of complications during and after vascular procedures. Although smoking has a proven effect on hemostasis, no literature is available on the effect of smoking on the activated clotting time (ACT), which is used to monitor the effect of heparin during noncardiac arterial procedures (NCAP). The aim of this study was to examine the effect of smoking on ACT values and the incidence of complications during the same admission or 30 day follow-up of NCAP.

**Materials and methods:**

A post hoc analysis of a prospective multicenter cohort study was performed. Patients older than 18 years, who underwent NCAP between December 2016 and April 2021, were enrolled. Patients were divided into 2 groups based on smoking status: never/former smokers and current smokers. Two heparin dosing protocols were used: an initial bolus of 5000 IU or 100 IU/kg bodyweight.

**Results:**

In total, 773 patients met the inclusion criteria. Five minutes after administration of 5000 IU of heparin, mean ACT values were 190 and 196 seconds for nonsmokers and smokers, respectively (p=0.078). After 100 IU/kg of heparin, mean ACT values were 229 and 226 seconds for nonsmokers and smokers, respectively (p=0.37). Incidence of complications in the whole study cohort was not significantly different for nonsmokers compared with smokers (arterial thrombo-embolic complication [ATEC] 4.7% vs 5.7% p=0.55; hemorrhagic complications 15% vs 18% p=0.29). In subgroup-analysis, a significant difference between smoking groups was found for hemorrhagic complications after open aneurysm repair (p=0.024). However, after adjusting for confounders, the difference between the smoking groups annulled.

**Conclusion:**

The results of this study suggest that smoking does not have a significant effect on ACT values or on the incidence of complications in NCAP. Large-scale studies are required to further analyze potential factors having an effect on the ACT and perioperative and postoperative complications, which could help individualize heparinization strategy.

**Clinical impact:**

There is high variance between patients in their response on administration of heparin, this is not yet fully understood. This study investigated the effect of smoking in a large prospective multicentre cohort. The results suggests that active smoking does not have an effect on the activated clotting time after administration of heparin. Also no significant effect of smoking could be found on the incidence of all registered complications. Monitoring of the effect of heparin remains important to provide patients with safe anticoagulation during vascular procedures.

## Introduction

Smoking is a well-known risk factor for developing vascular diseases such as abdominal aortic aneurysm, carotid artery stenosis, and peripheral arterial disease.^1–5^ In addition, graft failure, wound-related problems, amputation, and mortality are more common in smokers compared with nonsmokers during and after vascular procedures.^6–11^ This increase in perioperative complications such as amputations and death may be partly caused by inadequate anticoagulation during vascular procedures. In fact, smoking has a known effect on hemostasis. Previous research shows that smokers have a larger mean platelet volume, a higher Von Willebrand factor activity, and a shorter thrombin time, activated partial thromboplastin time, whole-blood clotting time and INR.^12–15^ Furthermore, smoking leads to inhibition of fibroblast migration, vasoconstriction and altered hemostasis.^6,16–19^ Therefore, it is conceivable that, compared with nonsmokers, smokers require more heparin during noncardiac arterial procedures (NCAP), to achieve the same level of anticoagulation.

To measure the antithrombotic effect of heparin during NCAP, the activated clotting time (ACT) can be used.^20^ The ACT is a point of care test to monitor the coagulation and is most accurate in high doses of heparin. Taking the effect of smoking on hemostasis and coagulation into account, the hypothesis was that smoking causes lower ACT values during NCAP. To this day, no study examined the effect of smoking on the ACT. In addition, limited studies have been performed investigating differences in incidence of complications between smokers and nonsmokers in NCAP. Therefore, the aim of this study was to examine the effect of smoking on ACT during NCAP and to investigate the incidence of arterial thrombo-embolic complications (ATECs), hemorrhagic complications and wound infections comparing smokers and nonsmokers.

## Materials and Methods

### Design and Patients

To examine the effect of smoking on ACT and the incidence of complications during and after NCAP, the current study was conducted as a post hoc analysis of a prospective collected database. For this study, the MANCO (official title: Measuring the ACT During Non-cardiac Arterial Procedures) registry was used. The MANCO registry is an ongoing, prospectively, multicenter registry for patients undergoing NCAP. The MANCO registry is registered at clinicaltrials.gov (identifier: NCT03426293). The protocol was evaluated by the Medical Ethical Committee Noord Holland. An opt-out procedure for informed consent was carried out. The participating hospitals were Dijklander Ziekenhuis, Hoorn, the Netherlands and Amsterdam UMC, location VU Medical Center, Amsterdam, the Netherlands.

Ongoing since the December 21, 2016, all patients older than 18 years undergoing elective NCAP in one of the aforementioned participating hospitals are registered in the MANCO registry. All patients underwent open, endovascular or hybrid NCAP.

In this study, patients were included having elective surgery between December 2016 and April 2021. Patients were excluded when having a known history of coagulation disorders. Additionally, for the current study, patients were excluded when the estimated glomerular filtration rate (eGFR) was lower than 30 mL/min (chronic kidney disease stage 4–5 based on KDIGO guidelines),^21^ when information concerning smoking was lacking or when the first dose of heparin was not according to one of the heparin dosing protocols.

### Heparin Dosing Protocols

Since the start of the MANCO registry, 2 protocols have been used. Patients received a standardized bolus of 5000 IU heparin (5000 IU group) or a body weight-dependent heparin dose of 100 IU/kg heparin (100 IU/kg group) with eventually additional heparin doses based on the ACT. The heparin bolus was administered after anesthetic induction and before arterial crossclamping. Local flushing of arteries was performed routinely using a sodium chloride solution with low dose of heparin.

### Anticoagulation Monitoring

The ACT was measured at the following time points using whole blood taken from an arterial catheter: before heparin administration (T0), 5 minutes after administering heparin (T1), and at 30 minute intervals thereafter (T2 and so on). At the completion of the procedure, a final ACT measurement was performed. At the surgeon’s discretion, protamine was administered to neutralize the effect of heparin. All ACT measurements were performed using the Hemostasis Management System Plus (HMS Plus, Medtronic Inc., Minneapolis, MN, USA), a point-of-care test.^22^ Delta ACT was calculated, being the difference between the baseline ACT and the ACT measured 5 minutes after heparin administration.

### Data Collection

All data were extracted from electronic health records (HiX ChipSoft at Dijklander Ziekenhuis and Epic electronic health records system at VU Medical Center) and were stored in the electronic data management platform Castor EDC. Besides ACT measurements, smoking history and complications, the following data regarding patient demographics were collected: sex, age, height, weight, body mass index (BMI, kg/m^2^), comorbidities, and medical history. Furthermore, data recorded included procedure details (type of procedure and anatomical details), preprocedural and postprocedural anticoagulants, and variables related to the surgical intervention.

### Smoking Status and Intervention Type

Concerning smoking history, patients were divided into 2 groups: nonsmokers (never smoked or former smokers) and smokers. Patients were classified as “former smokers” when they quit smoking at least 2 weeks prior to surgery. Additionally, patients were divided into 4 groups based on the type of intervention: (1) carotid endarterectomy (CEA); (2) aneurysm open procedure (open procedure of thoracoabdominal, abdominal or iliac aneurysm); (3) aneurysm endovascular procedure (endovascular or hybrid procedure of thoracoabdominal, abdominal or iliac aneurysm); and (4) procedure for peripheral arterial disease (PAD) (open, endovascular or hybrid procedure of peripheral arterial or aorta-iliac occlusive diseases).

### Primary and Secondary Endpoint

The primary endpoint was the relationship between smoking status and the mean ACT measured 5 and 30 minutes after the first heparin administration (T1) per heparin protocol group. The secondary endpoint was the incidence of complications during the same admission or within 30 day follow-up, comparing smoking status in total study cohort and per type of intervention subgroup.

Complications were (1) ATEC, which includes myocardial infarction, transient ischemic attack, cerebrovascular accident, deep venous thrombosis, pulmonary embolism, bowel ischemia, thrombo-embolic renal insufficiency, athero-embolism, spinal cord ischemia and graft thrombosis; (2) hemorrhagic complications, defined as European Multicenter Study on Coronary Artery Bypass Grafting (E-CABG) classification grade 1 or more (transfusion of platelets, plasma, or ≥2 units of RBCs); (3) wound infection; (4) renal insufficiency (not caused by thrombo-embolic occlusion); (5) major amputation or limp loss; and (6) death.

### Statistical Analysis

Statistical analyses were performed using the Statistical Package for the social Science (SPSS 27.0).^23^ The Kolmogorov–Smirnov test was used to assess normality in continuous variables. Continuous variables with a normal distribution were compared with the independent *t*-test, expressed as mean ± standard deviation. Skewed continuous variables were compared using the Mann-Whitney *U* test and expressed with median and interquartile range. Variables with a categorical character were analyzed using the Chi-Square test and were expressed with counts and percentages. Fisher's exact test for a 2 × 2 was used when frequencies for a cell were <5.

To adjust for potential confounding factors, a linear regression was used for the primary outcome (ACT after 5 minutes). To explore the association between smoking and postsurgical complications, a logistic regression model was used for univariate and multivariate analysis. Variables having a p-value < 0.2 in univariate analyses were included in multivariate analyses. A p-value < 0.05 was considered as statistically significant.

## Results

A total of 843 patients were enrolled in this study. Thirty patients were excluded due to an estimated Glomerular Filtration Rate (eGFR) below 30 mL/min, 4 patients were excluded because data concerning smoking history was lacking, 35 patients were excluded because of a protocol violation, and 1 patient was excluded due to not receiving heparin during the procedure. In total, 773 patients were evaluated. Of these, 324 patients received a standardized bolus of 5000 IU heparin and 449 patients received an initial dose of 100 IU/kg.

The patient demographics are shown in Table 1. In the 5000 IU group, smokers compared with nonsmokers were younger (68 vs 73 years; p≤0.001), less often male (70% vs 90%; p=0.036), and less often had cardiac disease in medical history (36% vs 47%, p=0.042). No other significant differences in medical history were found.

**Table 1.** Patient Demographics in the 5000 IU and 100 IU/kg Heparin Group.

Patient demographics	5000 IU group			100 IU/kg group		
	Smoking status			Smoking status		
	Yes (n=115)	No (n=209)	p-value	Yes (n=148)	No (n=301)	p-value
Age (years)	68 (12)	73 (10)	**<0.001**	70 (14)	74 (9)	**<0.001**
Sex (male)	80 (70)	167 (90)	**0.036**	103 (70)	217 (72)	0.58
Weight (kg)	79 (25)	80 (18)	0.26	74 (19)	80 (20)	**<0.001**
BMI (kg/m^2^)	26 (5.6)	26 (5.3)	0.21	24 (5.5)	26 (5.8)	**<0.001**
Medical history						
Hypertension	83 (72)	162 (78)	0.28	101 (68)	243 (81)	**0.003**
Hypercholesterolemia	39 (34)	66 (32)	0.67	42 (28)	126 (42)	**0.006**
Impaired renal function	7 (6.1)	13 (6.2)	0.96	3 (2)	18 (6)	0.062
Diabetes	25 (22)	52 (25)	0.71	32 (22)	76 (25)	0.31
Cardiac	41 (36)	99 (47)	**0.042**	49 (33)	132 (44)	**0.029**
TIA/CVA	32 (28)	66 (32)	0.48	50 (34)	114 (38)	0.40
Prior arterial intervention	47 (41)	71 (34)	0.22	53 (36)	101 (34)	0.64

Continuous data are shown as the median with interquartile range. Categorical variables are shown as count and percentage (%). Bold values indicate a p-value <0.05.

Abbreviations: BMI, body mass index; cardiac, acute infarction/ischemic, heart failure, cardiomyopathy, cardiomegaly, or atrial fibrillation; CVA, cerebrovascular accident; IU, international unit; kg, kilogram; n, number of patients; renal function impaired, estimated glomerular filtration rate 30< x<40 mL/min; TIA, transient ischemic attack.

Similar to the 5000 IU group, smokers were significantly younger than nonsmokers (median age 70 years vs 74 years; p<0.001) in the 100 IU/kg group. Smokers had a lower weight than nonsmokers (74 vs 80 kg; p<0.001), a lower BMI (24 kg/m^2^ vs 26 kg/m^2^; p<0.001), a lower incidence of hypertension (p=0.003), hypercholesterolemia (p=0.006) and cardiac history (p=0.029).

The preprocedural antithrombotic therapy is shown in Table 2. No significant differences between the groups were seen for any of the preprocedural antithrombotic therapies.

**Table 2.** Preprocedural Anticoagulants in the 5000 IU and 100 IU/kg Heparin Group.

Preprocedural anticoagulants^a^	5000 IU group			100 IU/kg group		
	Smoking status			Smoking status		
	Yes (n=115)	No (n=209)	p-value	Yes (n=148)	No (n=301)	p-value
None	6 (5.2)	24 (1.9)	0.100	5 (3.4)	11 (3.7)	0.88
Acetylsalicylic acid	31 (27)	64 (31)	0.49	41 (28)	72 (24)	0.39
Clopidogrel	30 (26)	61 (29)	0.55	59 (40)	111 (37)	0.54
Dual antiplatelet therapy	14 (12)	22 (11)	0.65	10 (6.8)	29 (9.6)	0.31
Vitamin K antagonist	10 (8.7)	31 (15)	0.11	13 (8.8)	30 (10)	0.69
DOAC	6 (5.2)	5 (2.4)	0.18	9 (6.1)	29 (9.6)	0.20
Combination	5 (4.3)	5 (2.4)	0.33	2 (1.4)	1 (0.3)	0.25

Categorical variables are shown as count and percentage (%). Bold values indicate a p-value <0.05.

Abbreviations: DOAC, direct oral anticoagulants; IU, international unit; n, number of patients.

In Table 3, the procedural types are summarized. When comparing between smoking groups, a higher percentage of CEA was performed in nonsmokers in the 5000 IU group (p=0.033). In the 100 IU/kg group, significant differences in percentage of open and endovascular aneurysm procedures performed was found, compared between smoking groups (p=0.023 and p=0.037 respectively).

**Table 3.** Type of Intervention in the 5000 IU and 100 IU/kg Heparin Group.

Type of intervention	5000 IU group			100 IU/kg group		
	Smoking status			Smoking status		
	Yes (n=115)	No (n=209)	p-value	Yes (n=148)	No (n=301)	p-value
CEA	12 (10)	41 (19)	**0.033**	32 (22)	88 (29)	0.087
Aneurysm open procedure	23 (20)	29 (14)	0.15	24 (16)	27 (9)	**0.023**
Aneurysm endovascular procedure	39 (34)	75 (36)	0.72	19 (13)	63 (21)	**0.037**
PAD	41 (36)	64 (31)	0.36	73 (49)	123 (41)	0.089

Categorical variables are shown as count and percentage (%). Bold values indicate a p-value < 0.05.

Abbreviations: CEA, carotid endarterectomy; IU, international unit; n, number of patients; PAD, peripheral arterial disease.

### Act

Mean ACT values, measured 5 and 30 minutes after heparin administration, are presented in Table 4. In the 5000 IU group, mean ACT values were 190 seconds for nonsmokers and 196 seconds for smokers (p=0.078). In the 100 IU/kg group mean ACT values were 229 and 226 seconds, respectively (p=0.37). No significant difference for ACT after 5 minutes was observed after adjusting for sex, BMI, age, hypertension, cardiac history, diabetes mellitus, chronic obstructive pulmonary disease, smoking and heparin dosing group (adjusted p=0.53). In addition, no difference was found in mean ACT values after 30 minutes. In the 5000 IU group, mean ACT values were 177 seconds for nonsmokers and 183 seconds for smokers (p=0.11). In the 100 IU/kg group mean ACT values were 212 and 210 seconds, respectively (p=0.66). There was also no difference between smokers and nonsmokers when the difference between baseline ACT and 5 minute ACT (delta ACT) was used as the outcome (crude p=0.72; adjusted p=0.90).

**Table 4.** Mean ACT in the 5000 IU and 100 IU/kg Heparin Group at Baseline (T0), 5 and 30 Minutes After Heparin Administration.

	5000 IU group			100 IU/kg group		
	Smoking status			Smoking status		
	Yes (n=115)	No (n=209)	p-value	Yes (n=148)	No (n=301)	p-value
Mean ACT (s) baseline	132	131	0.30	134	133	0.15
Mean ACT (s) after 5 minutes	196	190	0.078	226	229	0.37
Mean ACT (s) after 30 minutes	183	177	0.11	210	212	0.66
Mean delta ACT (s)	63	58	0.17	92	96	0.29

Delta ACT = the difference between the baseline ACT and the ACT measured 5 minutes after heparin administration.

Abbreviations: ACT, activated clotting time; IU. international unit; n. number of patients; s, seconds.

### Complications

Analysis on the incidence of complications during the same admission or 30 day follow-up was performed for the entire study cohort (Table 5). No significant difference between smoking groups was found regarding the incidence of ATEC, hemorrhagic complications, wound infection, renal insufficiency, major amputation/limb loss, and death.

**Table 5.** Incidence of Complications During 30 day Follow-up or During the Same Admission in Total Study Cohort.

Complications	Smoking status		p-value
	Yes (n=263)	No (n=510)	
ATEC	15 (5.7)	24 (4.7)	0.55
Myocardial infarction	1	5	
Transient ischemic attack	—	4	
Bowel ischemia	3	5	
Graft thrombosis			
Revision/redo-surgery	9	6	
Tissue loss/amputation	—	2	
Pulmonary embolism	—	1	
Spinal cord ischemia	2	1	
Hemorrhagic complications	47 (18)	76 (15)	0.29
Wound infection	22 (8.4)	25 (4.9)	0.056
Renal insufficiency	9 (3.4)	13 (2.5)	0.49
Major amputation/limb loss	6 (2.3)	1 (0.2)	**0.007**
Death	8 (3)	6 (1.2)	0.09

Categorical variables are shown as count and percentage (%). Bold values indicate a p-value <0.05.

Abbreviations: ATEC, arterial thrombo-embolic complication; E-CABG≥1, hemorrhagic complications; n, number of patients.

In addition, the complication rates were assessed within subgroups, defined by intervention type (Table 6). A significant difference between smoking groups was found in hemorrhagic complications after open aneurysm procedure (30% in smokers vs 52% in nonsmokers, p=0.024).

**Table 6.** Incidence of Complications During 30 Days of Follow-up or During the Same Admission in Total Study Cohort Per Type of Intervention.

Type of intervention	Smoking status		p-value
	Yes (n=44)	No (n=129)	
CEA			
ATEC	1 (2.3)	4 (3.1)	1.0
Hemorrhagic complications	0 (0)	3 (2.3)	0.57
Wound infection	0 (0)	0 (0)	—
Aneurysm open procedure	47	56	
ATEC	5 (11)	6 (11)	0.99
Hemorrhagic complications	14 (30)	29 (52)	**0.024**
Wound infection	0 (0)	0 (0)	—
Aneurysm endovascular procedure	58	138	
ATEC	3 (5.2)	3 (2.2)	0.36
Hemorrhagic complications	6 (10)	8 (5.8)	0.26
Wound infection	1 (1.7)	0 (0)	0.30
PAD	114	187	
ATEC	6 (5.3)	11 (5.9)	0.82
Hemorrhagic complications	27 (24)	36 (19)	0.36
Wound infection	22 (19)	25 (13)	0.17

Categorical variables are shown as count and percentage (%). Bold values indicate a p-value <0.05.

Abbreviations: ATEC, arterial thrombo-embolic complication; CEA, carotid endarterectomy; E-CABG ≥ 1, hemorrhagic complications; n, number of patients; PAD, peripheral arterial disease.

Univariate and multivariate analyses were performed to assess the possible effect of confounding factors on the occurrence of hemorrhagic complications after open aneurysm procedure (Table 7). Univariate analysis showed a considerable effect (p<0.2) of BMI, age and smoking. However, after correction for confounders, no difference was found between the smoking groups for hemorrhagic complications.

**Table 7.** Univariate and Multivariate Analyses on the Incidence of Hemorrhagic Complication in the Open Aneurysm Group.

Variables	Univariate (n=196)			Multivariate		
	p-value	Odds ratio	CI	p-value	Odds ratio	CI
Sex	0.91	1.1	0.40–2.8			
BMI^a^	**0.16**	**0.94**	**0.86**–**1.0**	0.33	0.96	0.87–1.0
Age^a^	**0.004**	**1.1**	**1.0**–**1.2**	**0.028**	**1.1**	**1.0**–**1.1**
Hypertension	0.82	0.90	0.38–2.1			
Cardiac history	0.67	0.84	0.38–1.9			
DM	0.80	0.88	0.31–2.4			
COPD	0.22	1.9	0.69–5.0			
Peak ACT^a^	0.33	1.0	1.0–1.0			
Smoking	**0.026**	**0.40**	**0.18**–**0.89**	0.11	0.49	0.21–1.2

Bold values indicate a p-value <0.2 in univariate analyses and a p-value <0.05 in multivariate analyses.

Abbreviations: BMI, body mass index; cardiac history, heart failure, ischemic heart disease/coronary artery disease or atrial fibrillation; CI, confidence interval; COPD, chronic obstructive pulmonary disease; DM, diabetes mellitus; Multivariate, multivariate analyses; Univariate, univariate analyses.

a Continuous variable.

## Discussion

In modern-day NCAP, measuring the ACT is slowly becoming a standard operating procedure to monitor the anticoagulant effect of heparin. In the search for an optimal heparin strategy, this study was conducted to analyze the influence of smoking on ACT. This topic is also of current interest in cardiac interventions, as a prospective study on differences in ACT values between smokers and nonsmokers during percutaneous transluminal coronary angioplasty is currently being conducted.^24^

The current study showed that smoking, comparing smokers and nonsmokers, does not have a significant effect on the ACT measured 5 minutes after the administration of heparin. No significant difference was found in the 5000 IU heparin group, nor in the 100 IU/kg heparin group. Furthermore, no significant effect was found in the incidence of complications within 30 day follow-up or during the same admission, comparing smoking status in the whole study cohort.

Despite ACT measurements being performed in NCAP for more than 40 years, no studies have been performed investigating the effect of smoking on ACT values. Nonetheless, 2 studies found an effect of smoking on a coagulation screening test. In one study, smokers tended to have a shorter prothrombin time and international normalized ratio compared with nonsmokers.^14^ In the second study, male smokers had a significantly shorter thrombin time compared with male nonsmokers.^13^ Due to these studies, the hypothesis of this present study was that smoking causes lower ACT values. However, in the current study, no effect of smoking on ACT was found. This might be caused by a difference in patient characteristics and the number of included patients, as previous studies included 92 and 200 young healthy subjects from Kuwait and Sudan, while the current study included 773 elderly Western patients with vascular disease. Age is considered as a potential influential factor, as previous research showed an association between age and an increased risk of hemorrhagic complications.^25^ In addition, univariate analyses conducted within in the current study showed that age, smoking and BMI influence the incidence of hemorrhagic complications in open aneurysm repair. This effect was annulled for smoking and BMI in multivariate analysis, but not for age.

No literature is available on the effect of smoking on ATECs and hemorrhagic complications in NCAP to compare results. However, a few articles are available in which the effect of smoking on the incidence of other complications in NCAP is examined.

A meta-analysis showed that smoking was associated with a 2.35 fold increase in graft occlusion in PAD procedures.^9^ It should be noted that follow-up in this meta-analysis ranged from 6 months to 10 years, and follow-up in this study was 30 days, comparing these studies is inconvenient.

In a systematic review on general surgery, wound healing was found to be impaired in patients who smoke.^6^ Although cessation of smoking restores the healing capacity within four weeks, a negative effect on healing remains.^6^ In our cohort no effect of smoking on wound healing was found.

In regard to mortality, no significance could be determined because of low mortality rates in this study. In a review of Marques-Rios et al., smoking was a predictor of long-term mortality in endovascular repair of abdominal aortic aneurysms.^8^ In addition, Kim et al.^11^ showed that smokers were at increased risk for death within 30 days after carotid endarterectomy.

The studies mentioned indicate that smoking seems to have an effect on complications during and after vascular surgery. However, this study only assessed the complications 30 days after surgery while aforementioned complications occurred after a longer follow-up time. Therefore, it is recommended that future studies explore the impact of smoking on complications during both short-term and long-term follow-up periods.

One of the strengths of this current study is the use of a multicenter database, which results in a better external validity. Furthermore, the 2 most common worldwide used heparin dosing protocols (initial dose of 5000 IU of heparin and initial dose of 100 IU/kg heparin) were included in this study. In addition, all ACT measurements were performed using the Hemostasis Management System Plus (HMS Plus, Medtronic Inc., Minneapolis, MN, USA). As a result, the ACT values measured are reliable and therefore comparable within this study. This is important, since significant variability in ACT values between different brands of ACT devices has been reported.^26–29^

Some limitations should be noted. Although a large study cohort was used, the sample size was too small for accurate subgroup analysis due to low complication incidence rates. In addition, due to inconsistent registration in medical records, smoking history was not further defined by intensity (packs/day), duration (years), pack-year history, or detailed cessation time. Furthermore, no nicotine or cotinine test was used to verify the self-reported nonsmoking status. Lastly, having a known history of coagulation disorders was a reason for exclusion. However, despite literature shows reduced responsiveness of ACT to heparin when antithrombin III level decreases, patients were not actively screened for antithrombin III levels.^30^

## Conclusion

This study suggests that active smoking does not have an effect on the ACT after administration of a standardized bolus of 5000 IU of heparin or a body weight-dependent heparin dose of 100 IU/kg. No significant effect of smoking could be found on the incidence of all registered complications.
